# A Retrospective Study of Ultrasound-Guided Pericapsular Nerve Group Block With Dexamethasone: An Excellent Option for Early Mobility Following Total Hip Replacement Surgery

**DOI:** 10.7759/cureus.32515

**Published:** 2022-12-14

**Authors:** Nishu Gupta, Samaresh Das, Nilay Chatterjee, Manish Munjal

**Affiliations:** 1 Trauma and Orthopaedics, Yeovil District Hospital, Yeovil, GBR; 2 Anaesthesia, Yeovil District Hospital, Yeovil, GBR; 3 Anaesthesia, ManglamPlus Medicity Hospital, Jaipur, IND

**Keywords:** enhanced recovery, post-operative mobility, regional analgesia, peng block, total hip replacement

## Abstract

Background

Severe postoperative pain and immobility increase the length of hospital stay and immobility-related life-threatening complications after total hip replacement (THR). Pericapsular nerve group (PENG) block is a recent addition to pain management of neck of femur (NoF) fracture, the use of which has been incorporated into THR as alternative analgesia or as an adjunct with other regional analgesia techniques. The present study primarily aims to assess postoperative mobility. Secondary outcomes measured were the length of hospital stay, pain score, opioid consumption, and side effects.

Methods

This is a retrospective study of 50 patients who underwent primary THR. Twenty-eight patients received PENG block after spinal anesthesia (PENG Group), seven patients had general anesthesia (GA) with patient-controlled analgesia (PCA) postoperatively (PCA Group), and the remaining 15 received spinal anesthesia with fascia iliaca block (FIB Group). Mobilization was attempted in all patients (ability to stand and walk a few steps with a walker) 10 hours after the end of surgery. Data was collected for average postoperative pain score, time of mobilization, total opioid consumption (till discharge from the hospital), opioid-related side effects, and time of discharge.

Results

Mobilization was attempted in all patients 10 hours after the end of the surgery, irrespective of their anesthetic technique. In the PENG Group, 26 patients (n=28) could be mobilized after the first 10 hours without opioids. The total morphine requirement until discharge was significantly less in the PENG Group of patients compared to the FIB and GA+PCA patients. The average time of discharge (hours) from the hospital (22.1+/-4.9) was also significantly lower in the PENG Group compared to all other groups (31.7 +/- 3.4, p=<0.01). The average postoperative pain score was significantly low in the PENG Group within the first 48 hours.

Conclusion

The PENG block helps in early mobilization and enhanced recovery after THR.

## Introduction

One of the most effective treatments for severe hip osteoarthritis is total hip replacement (THR), which improves the quality of life [1]. Despite the improvement in the quality and functional ability, immediate postoperative pain and immobility are the two factors that increase the duration of hospitalization and complications following THR [2]. Among the postoperative complications of THR, the most life-threatening are deep vein thrombosis and pulmonary embolism, which are directly related to lack of mobility. Various anaesthetic and analgesic methods are used for THR. These include general anesthesia (GA), patient-controlled anesthesia (PCA), spinal anesthesia with opioids and lumbar epidural, among others. Also used are nerve blocks, e.g., lumbar plexus block, fascia iliaca block (FIB), femoral, obturator, and sciatic nerve block. Opioids are associated with various side effects, even when used through neuraxial (spinal or epidural) routes. At times, neuraxial anesthesia could also be associated with complications like spinal hematoma, headache, prolonged hospitalization due to motor weakness, and delayed mobilization. Regional nerve blocks, e.g., lumbar plexus block, and compartment blocks, e.g., FIB, femoral, obturator, and sciatic nerve block, might cause significant muscle weakness (quadriceps weakness) that delays mobilization and hospital discharge.

A recent addition to the armamentarium for managing acute pain in the fractured neck of the femur (NoF) patients is the ultrasound-guided pericapsular nerve group (PENG) block, first described by Giron-Arango et al. [3]. PENG block primarily targets sensory innervations of the anterior hip capsule by blocking the articular branches of the femoral, obturator, and accessory obturator nerves. Recently, the use of PENG block has been adopted in routine hip arthroplasty as alternative analgesia or with other routine regional analgesia techniques or opioids [4]. We report a retrospective series of 50 patients who underwent THR (indication: painful severe osteoarthritis), most of whom received the PENG block. The primary objective of the study was to review early postoperative mobility in patients with PENG blocks as compared with other modes of anesthesia and analgesia. The secondary objective was to compare the length of hospital stay, the pain score, opioid consumption, and side effects.

## Materials and methods

This retrospective study was conducted on 50 patients aged 60-80 years who underwent primary THR (posterior approach) in a tertiary care hospital between January 2022 and June 2022. Among 50 patients, 28 received PENG block after spinal anesthesia (L4-5 level, in the sitting position and using bupivacaine heavy 2.5 ml, (PENG Group)), and seven received GA with PCA morphine or oxycodone postoperatively (PCA Group) as they refused regional anesthesia. The remaining 15 patients received spinal anesthesia (L4-5 level, in the sitting position and using bupivacaine heavy 2.5 ml) with fascia iliaca block (FIB Group, 20 ml of local anesthetic, 0.5% levobupivacaine). Written consent was obtained from the individual patients for the PENG and FIB blocks.

All the operations were performed by the same surgical and anaesthetic team. In addition to regional and neuraxial anesthesia, wound infiltration with 50 ml (0.25% levobupivacaine) was done in all the patients after the surgical procedure. Therefore, wound infiltration essentially helped to eliminate pain from the incision site.

The numeric rating scale (NRS) scale of 0-10 (0=no pain, 10=worst perceivable pain) for pain was recorded postoperatively at the orthopaedic postoperative unit by a qualified nurse at 1, 6, 12, 24, and 48 hours. Mobilization was attempted in all patients (the ability to stand and walk a few steps for the first time after surgery with the help of a walker) after 10 hours of surgery.

All the patients included in the study received similar multimodal analgesia with paracetamol 1000 mg four times a day and ketorolac 30 mg (for 48 hours only) three times a day in addition to nerve block or PCA (in patients who received GA). In addition, oral morphine 5-10 mg (or morphine equivalent doses of other opioids) was prescribed for all breakthrough (NRS more than 6) pain. All the patients were observed for 48 hours for local anesthesia toxicity, hematoma, and neurological injury before discharge.

PENG block technique

**Figure 1:**
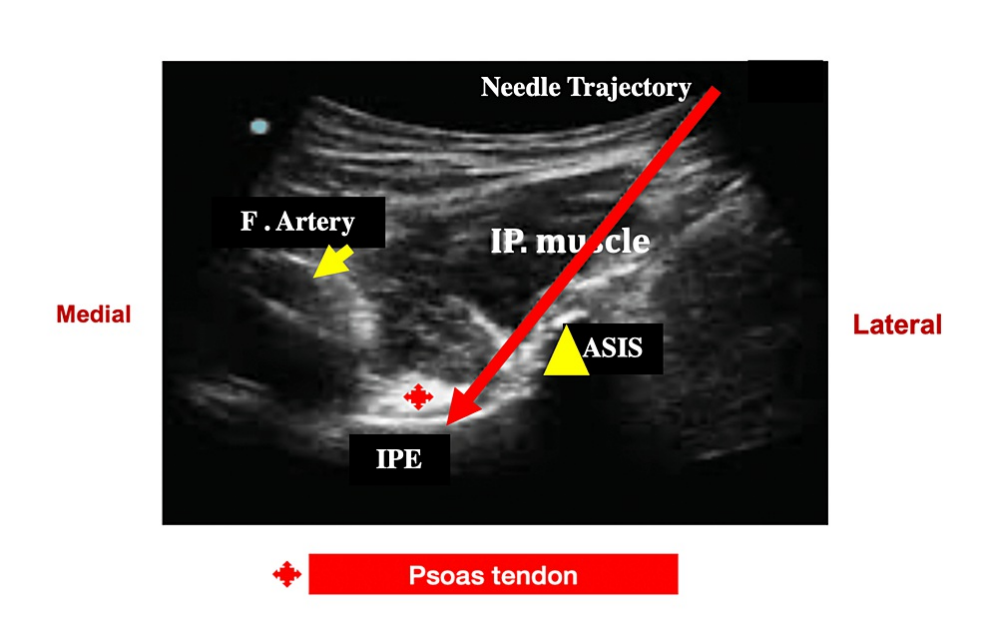
PENG block under ultrasound guidance PENG: pericapsular nerve group; ASIS: anterior superior iliac spine; IPE: iliopubic eminence.

PENG block involves injecting local anesthetics in the musculofascial plane between the psoas muscle and the superior pubic ramus under ultrasound guidance (Figure 1). We performed the PENG block using the curvilinear transducer (3-8 MHz), placing the patient in a supine position (ultrasound machine - Sonosite SII; Fujifilm Sonosite Europe, Amsterdam, Netherlands). Under strict sterile precautions, the transducer was placed parallel to the inguinal crease at the anterior superior iliac spine (ASIS) level. Then, the transducer was gradually moved to a caudal direction to identify the anterior inferior iliac spine (AIIS) and iliopubic eminence (IPE). The psoas muscle and tendon (hyperechoic structure) were identified just above the IPE. The femoral vessels were far medial to the psoas tendon and IPE. A 100-mm, 21G echogenic needle (Pajunk GmbH Medizintechnologie, Geisingen, Germany) was introduced using an in-plane technique from lateral to medial direction. The needle path was visualized throughout, and 20 ml of local anesthetic (0.5% levobupivacaine) with 8 mg of preservative-free dexamethasone was deposited under the psoas tendon after negative aspiration for blood.

Patients receiving GA were induced with propofol/rocuronium and fentanyl as analgesics (none received any long-acting analgesic during the procedure). Anesthesia was maintained with air/oxygen (65:35), sevoflurane (MAC 1.2), and intermittent boluses of rocuronium. Postoperatively, PCA morphine was started immediately after the operation. Fifteen patients received ultrasound-guided FIB block after spinal anesthesia. After negative aspiration, they all received 20 ml of local anesthetic (0.5% levobupivacaine) with 8 mg of preservative-free dexamethasone. None of the patients in either group received intravenous dexamethasone during the procedure.

## Results

Demographic profiles were comparable between patients allocated to different groups (Table 1). Mobilization was attempted in all patients by 10 hours following surgery, irrespective of their anesthetic technique. In the PENG Group, 26 patients (n=28) could be mobilized after the first 10 hours without opioids. Overall PENG Group patients required significantly less opioids compared to other groups (Table 2). The total morphine requirement until discharge was significantly less in PENG Group patients compared to FIB and GA+PCA patients (Table 2). The average time of discharge (hours) from the hospital (22.1+/-4.9) was also significantly lower in the PENG Group compared to all others (31.7 +/- 3.4, p=<0.01) (Table 2). The average postoperative pain score was significantly low in the PENG Group within the first 48 hours (Table 3). Opioid-related side effects were least in the PENG Group.

**Table 1:** Demographic data PENG: pericapsular nerve group; FIB: fascia iliaca block; GA: general anesthesia; PCA: patient-controlled analgesia.

	PENG Group	FIB Group	GA+PCA Group	p
Number	28	15	7	
Gender (M: F)	13:15	8:7	5:2	NS
Age (years) (mean+/-SD)	70.9 +/- 10.8	69.5 +/- 11.9	67.5 +/- 8.5	NS

**Table 2:** Total opioid consumption, mobilization, and time of discharge from the hospital PENG: pericapsular nerve group; FIB: fascia iliaca block; GA: general anesthesia; PCA: patient-controlled analgesia.

	PENG Group	FIB Group	GA+PCA Group	p (PENG vs. FIB)	p (PENG vs. GA+PCA)	p (FIB vs. GA+PCA)
Opioid (morphine/equivalent) consumption in mg	0.7 +/- 1.6	5.8 +/- 1.1	81.4 +/- 23	<0.001**	<0.001**	<0.001**
No of patients mobilized at 10 hours after surgery without additional opioids	26/28 (92.8%)	4/15 (26.6%)	0/7 (0%)	<0.001**	<0.001**	<0.001**
length of hospital stay from surgery in hours	22.4 +/- 4.7	25.6 +/- 0.8	34.3 +/- 8.6	0.076	<0.001**	0.02*

**Table 3:** Post-operative pain score PENG: pericapsular nerve group; FIB: fascia iliaca block; GA: general anesthesia; PCA: patient-controlled analgesia.

Time (after surgery)	PENG Group	FIB Group	GA+PCA Group	p (PENG vs. FIB)	p (PENG vs. GA+PCA)	p (FIB vs. GA+PCA)
1 hour	0.8 +/- 0.9	1.5 +/- 1.3	3.6 +/- 2.2	0.06	<0.001**	0.007**
6 hours	1 +/- 0.9	1.6 +/- 1.2	3.1 +/- 1.2	0.07	<0.001**	0.014*
12 hours	1.2 +/- 1.4	2.2 +/- 1.6	3.8 +/- 2	0.02*	0.0001**	0.045*
24 hours	1.5 +/- 1.5	2.4 +/- 1.5	4.4 +/- 1.9	0.069	0.0017**	0.011*
48 hours	2.5 +/- 1.3	3.2 +/- 1.1	4.6 +/- 1.4	0.063	0.0005**	0.014*

## Discussion

Hip surgery is the most commonly performed on elderly patients. Early mobilization and timely physical therapy after hip surgery are significant factors for a successful surgical outcome. However, oversedation with strong opioid analgesics may interrupt effective communication between the patient and the health care team, which can delay early mobilization and rehabilitation [5]. Importantly, delayed mobilization is one of the leading causes of prolonged hospital stay, which adds to the overall burden on the health care system. In addition, the use of opioids is also associated with morbidity in terms of side effects, e.g., nausea, vomiting, dizziness, abdominal distension, respiratory depression, and delirium in the older population.

FIB has been traditionally used for fracture NoF and hip surgeries. The spread of local anesthesia after FIB was studied with MRI, and it was found that local anesthetics spread in the cephalad direction [6]. Another case study revealed that FIB does not consistently cover the obturator nerve [7]. Therefore, many times, additional analgesia is needed after the FIB block. FIB mainly blocks the femoral nerve. As a result, there will be quadriceps weakness when a large volume of local anesthetic is administered. Quadriceps weakness can delay ambulation and increase the likelihood of falls in the postoperative period.

The hip joint is a synovial ball and socket joint, and the head of the femur forms the ball. The area is supplied by both the lumbar (L1-L4) and sacral (L4-S4) plexus. Short et al. described that the anterior hip capsule is innervated by mainly articular branches of the femoral and obturator nerve, providing a specific sensory supply [8]. In contrast, the medial capsule gets its sensory supply from the accessory obturator nerve [9]. However, posterior and inferior capsules have no sensory fibres [6]; mainly, mechanoreceptors are present there [10]. Gerhardt et al. performed PENG block in a cadaver [11]. They found contrast spreading in the anterior hip capsule and along the articular branches of the femoral, obturator, and accessory obturator nerves. Therefore, we can theoretically postulate that the PENG block could provide complete hip analgesia without blocking motor components because it mainly blocks the articular branches.

Sensory blockage of articular branches to the hip joint is essential for adequate analgesia after a hip surgery. Recently, different studies revealed the benefits of PENG block after hip fractures and THR [3,12]. For example, Pagano et al. found that the PENG block is very effective following hip fracture, mainly for positioning before spinal anaesthesia [13]. They also found its effectiveness for postoperative analgesia without affecting motor function. 

In our retrospective study, 28 patients received PENG block using levobupivacaine with dexamethasone after the spinal anaesthesia. Twenty-six patients were comfortable and did not require any analgesic during early mobilization within 10 hours of surgery. Two patients with the PENG group received additional rescue analgesia (oral morphine) before starting mobilization. The total dose of opioids requirements within the first 48 hours in the PENG group was also minimal compared to FIB and GA+PCA groups. Twenty-five patients from the PENG group were discharged within the first 48 hours, whereas only two patients from the FIB group were discharged within 48 hours of surgery. The main reason for the delayed discharge was pain, opioid-related side effects, and delayed mobilization.

Our findings were consistent with a recently published randomized control trial by Pascarella et al [14]. They concluded that PENG block is an effective analgesic technique for early postoperative analgesia. In addition, they found it very useful for fast-track hip surgery as it provided optimal analgesia, faster motor recovery, and fewer opioid requirements. Finally, in a retrospective case series, Kukreja et al. found it very useful for primary hip arthroplasty as a motor-sparing effect of the PENG block [15].

However, none of the previous studies added adjuvants with local anesthesia while administering the PENG block. In our study, we have added dexamethasone (as an adjuvant analgesic) with levobupivacaine. That may be one of the reasons for the extended analgesic effect and less use of opioids in the postoperative period. Many of our results corroborate with the existing literature. Thus it proves the already existing knowledge about the usefulness of PENG block in hip surgeries.

The limitations of the study were that this is a retrospective, single-centre study which used a small sample size. Also, there was no randomization involved.

## Conclusions

The PENG block with dexamethasone adjuvant is very effective for enhanced recovery after THR. Early mobilization is known to reduce complications in hip surgery. PENG block with dexamethasone provides adequate analgesia for early mobilization and is technically easy to perform in the supine position after spinal analgesia. The PENG block with dexamethasone adjuvant also leads to a reduction in opioid use and hence, a reduction in associated complications. The postoperative pain score in patients with the PENG block with dexamethasone adjuvant was also low. All these factors combined to reduce the length of stay and facilitated early discharge. Randomized controlled trials may be needed to compare the analgesic effect, duration, and usefulness for early mobilization when additives such as dexamethasone are used along with local anesthetics versus when only local anesthetics are used for the block.
